# Developments in Nanopatterning of Graphene; Toward Direct Writing

**DOI:** 10.1002/adma.202513264

**Published:** 2025-11-20

**Authors:** Szymon Abrahamczyk, Ondřej Sakreida, Alicja Bachmatiuk, Gražyna Simha Martynková, Mark H. Rümmeli

**Affiliations:** ^1^ EBEAM Centre CNT CEET VŠB TUO Ostrava 708 00 Czechia; ^2^ The Leibniz Institute for Solid State and Materials Research Dresden (IFW Dresden) Helmholtzstr. 20 D‐01069 Dresden Germany; ^3^ Soochow Institute for Energy and Materials InnovationS (SIEMIS) Optoelectronics and Energy and Collaborative Innovation Center of Suzhou Nano Science and Technology Key Laboratory of Advanced Carbon Materials and Wearable Energy Technologies of Jiangsu Province School of Energy Soochow University Suzhou 215006 China; ^4^ Faculty of Chemistry Wroclaw University of Science and Technology Wybrzeze Wyspiarskiego 27 Wroclaw 50‐370 Poland; ^5^ Nanotechnology Centre, CEET VŠB‐Technical University of Ostrava Ostrava 708 00 Czech Republic

**Keywords:** direct write, focused electron beam‐induced deposition (FEBID), focused ion beam milling (FIBM), das injection system (GIS), graphene nanopatterning, laser‐induced graphitisation, polymer‐to‐graphene conversion (P2G)

## Abstract

Graphene, with its exceptional electronic, mechanical, and thermal properties, remains a cornerstone material for next‐generation nanoelectronics. However, conventional lithographic approaches to graphene patterning are fraught with challenges, including contamination, alignment complexity, and scalability constraints. This review critically examines the evolving landscape of direct‐write graphene technologies, focusing on forefront strategies such as focused electron beam‐induced deposition (FEBID), polymer‐to‐graphene (P2G) conversion, focused ion beam (FIB) modification, and laser‐assisted graphitisation. These techniques represent a departure from traditional top‐down or transfer‐based methods by enabling bottom‐up, spatially resolved patterning without intermediary masking steps. Particular attention is devoted to the physicochemical mechanisms that underlie electron‐ and photon‐mediated graphitisation, the role of precursor chemistry and substrate interactions, as well as the influence of beam parameters on sp^2^‐carbon content and structural ordering. The review further delineates the limitations intrinsic to current methodologies, including partial graphitisation, resolution fidelity, and hardware constraints, and proposes a roadmap to achieve truly “direct” graphene writing. This includes in situ processing under controlled environments, advanced beam control systems, and the adoption of catalytic and graphitizable precursors. Collectively, this work provides a comprehensive foundation for the rational design of next‐generation nanofabrication protocols and underscores the transformative potential of direct‐write techniques in enabling scalable, high‐fidelity graphene‐based devices.

## Graphical abstract

Overview of principal direct‐write graphene patterning methodologies including focused electron beam induced deposition (FEBID), laser induced graphitisation (LIG), focused ion beam (FIB), scanning tunneling microscopy (STM) and atomic force microscopy (AFM)

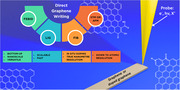

## 1 Introduction

### 1.1 What is Graphene?

Graphene was first confirmed and isolated as a stable free‐standing structure by Novoselov, Geim, Morozov, Jiang, Zhang, Dubonos, Grigorieva, and Firsov in 2004 using the famous Scotch tape method.^[^ ^1^, ^2^, ^3^ ^]^ This discovery initiated a rapid expansion of research across physics, materials science and engineering, leading to extensive studies on its applications in electronics, nanotechnology, and energy storage.^[^ ^4^, ^5^ ^]^ The non‐exhaustive list of notable developments would include advanced synthesis techniques such as chemical vapour deposition (CVD), epitaxial growth on SiC, pyrolysis, liquid phase exfoliation (LPE), polymer‐to‐graphene conversion (P2G), reduction of graphene oxide,^[^ ^6^ ^]^ elucidation of the electronic and quantum properties of graphene (graphene transistors, quantum effects, high‐frequency electronics),^[^ ^7^ ^]^ introduction into materials science and engineering (composites, energy storage, thermal management),^[^ ^8^ ^]^ biomedical and environmental applications (biosensors, drug delivery, water filtration and desalination)^[^ ^9^ ^]^ etc.

Graphene's unique structure confers remarkable properties, including high electrical conductivity, mechanical strength, and thermal stability, making it ideal for nanotechnology applications.^[^ ^2^ ^]^ A single undoped layer of graphene (SLG) is a zero‐bandgap material allowing a conduction of massless Dirac fermions making it an excellent conductor with electrical conductivity of ≈10^5^ S m^−1^, comparable to that of metallic silver in bulk ≈6.2 × 10^7^ S m^−1^.^[^ ^2^, ^10^ ^]^ The deterioration of the conductivity of metals in nanoscale due to quantum size effects and surface scattering of the electrons (down to < 10^4^ S m^−1^) provides single‐layer pristine graphene a significant benefit for application in nanometric‐scale electronics as a conductor.^[^ ^11^, ^12^ ^]^ However, this advantage is highly dependent on the quality and fabrication method of the graphene used.

The quality of graphene often refers to the number of layers, the presence of defects, the chemical uniformity, and the size of the graphene domain.^[^ ^13^, ^14^ ^]^ For example, a double‐layer graphene has an order of magnitude lower electrical conductivity (10^4^ S m^−1^) than that of SLG.^[^ ^15^ ^]^ While in SLG the conduction (CB) and valence bands (VB) are touching at a single Dirac point, the graphene bilayer exhibits interlayer interactions (van der Waals and π − π spin‐orbit coupling) in effect creating a finite bandgap (< 0.2 eV), making the electrons behave much more as particles with an effective mass.^[^ ^16^ ^]^ Further stacking and defects will also affect the conductivity of the synthesised graphene, making this material difficult to process.^[^ ^17^, ^18^, ^19^ ^]^

**Figure 1:**
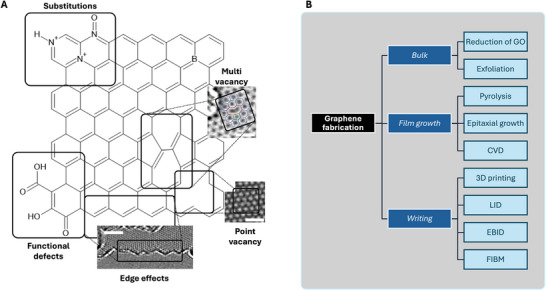
A) Simplified chemical structure of a graphene sheet, and examples of possible doping/defect states. All the image have a white scale bar representing 2 nm. The edge effect TEM image is reproduced with permission.^[^ ^20^ ^]^ Copyright 2009, American Chemical Society. The point vacancy TEM image is reproduced with permission.^[^ ^21^ ^]^ Copyright 2013, American Chemical Society. The double vacancy TEM image is reproduced with permission.^[^ ^22^ ^]^ B) Recognised processes used for graphene fabrication. Interpretation of similar figures from ref. [23] and ref. [17]

Graphene defects are typically classified into several types: point defects such as vacancies,^[^ ^21^, ^24^ ^]^ interstitial defects, substitutions,^[^ ^25^, ^26^, ^27^ ^]^ or adatoms;^[^ ^28^ ^]^ line defects such as grain boundaries^[^ ^29^ ^]^ and dislocations;^[^ ^29^, ^30^ ^]^ plane defects such as multilayer regions, folds, and wrinkles;^[^ ^31^ ^]^ structural distortions, including Stone–Wales defects,^[^ ^32^ ^]^ superlattice defects,^[^ ^33^ ^]^ ripples, and corrugations; and chemical and functional defects such as oxidised and hydrogenated areas, as well as edge defects.^[^ ^20^ ^]^ These dopants and defect states will significantly affect the electronic structure of graphene and introduce a band gap.^[^ ^13^, ^34^ ^]^ **Figure** **1** illustrates how this doping might appear in a graphene sheet. These, depending on the situation, can be considered as unwanted defects that deteriorate the quality of graphene, or dopants that make graphene a material with a highly tunable bandgap.^[^ ^13^, ^35^ ^]^

Chemical doping can be introduced into graphene by incorporating heteroatoms (B,^[^ ^36^ ^]^ N,^[^ ^36^, ^37^, ^38^ ^]^ P,^[^ ^39^, ^40^ ^]^ O,^[^ ^41^ ^]^ S,^[^ ^42^ ^]^ F,^[^ ^43^ ^]^ Cl,^[^ ^44^, ^45^ ^]^ Al,^[^ ^27^ ^]^ Co,^[^ ^46^ ^]^ H,^[^ ^47^, ^48^ ^]^ Si, Rb^[^ ^49^ ^]^) into the graphene lattice that donate or accept electrons, functionalisation of graphene (oxidation to rGO) that disrupts the graphene's *sp* ^2^ hybridisation, or even adsorption of metal atoms, particles, or even molecules that would transfer their charge to the graphene surface. The chemical doping gives a lot of control over the type and degree of doping, allowing bandgap tuning from 0 to 2.9 eV.

However, structural changes such as single or multiple vacancies that allow for chemical homogeneity are not currently difficult to introduce with controlled processes. These can alter the electronic properties of graphene by the introduction of strains, rotations, and lattice mismatches.^[^ ^50^ ^]^

The last but not least type of graphene quality parameter is its graphene domain size. Small graphene domains have orders of magnitude lower carrier mobilities as a result of phonons and impurity scattering contributing to resistivity, whereas in large graphene domains the transport is more ballistic.^[^ ^1^ ^]^ The small domains below 10 nm can even experience quantum confinement effects shifting the Dirac cone to opening a bandgap.^[^ ^51^ ^]^ Furthermore, the alignment and orientation of graphene domains, especially in polycrystalline films, can lead to the formation of grain boundaries and twisting between domains, significantly impacting both charge mobility and structural strength. Depending on the twist angle, these twisted domains can display moiré superlattice patterns, giving rise to novel electronic effects, including flat bands and correlated insulating phases, as seen in twisted bilayer graphene. Consequently, the fabrication of large‐area, single‐crystal graphene is crucial for high‐performance electronic and optoelectronic applications. These monocrystalline graphene sheets reduce grain boundary scattering and maintain graphene's inherent properties, such as its extremely high carrier mobility and linear band structure. Significant effort has been invested in refining CVD methods to manage nucleation density and growth kinetics, enabling centimetre‐scale single‐crystal domains.

**Figure 2:**
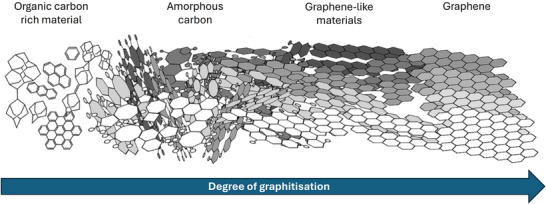
A schematic representation of degree of graphitisation. The image is adapted under the terms of the CC BY 4.0 license.^[^ ^52^ ^]^ Copyright 2020, The Authors.

**Figure 3:**
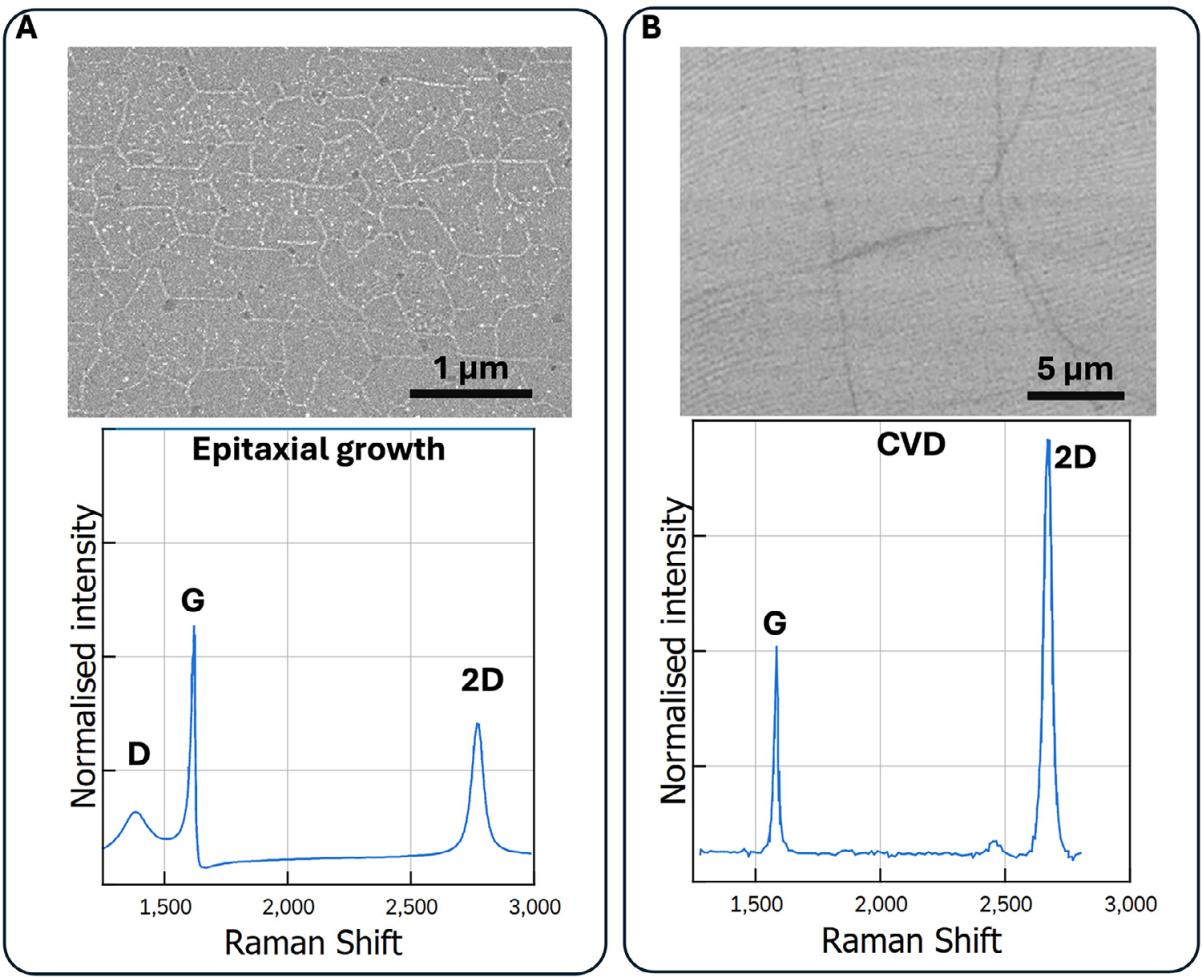
A) SEM image and a Raman spectrum of graphene grown using epitaxial growth on SiC. Reproduced with permission.^[^ ^78^ ^]^ Copyright 2020, Elsevier. B) SEM image and a Raman spectrum of a monolayer graphene sheet grown using CVD and transferred onto Si. Reproduced with permission.^[^ ^79^ ^]^ Copyright 2022, Wiley. The Raman spectra were reproduced and digitised from plots in the citations^[^ ^78^, ^79^ ^]^

The quality of graphene can be quantified using multitechnique approach combining structural characterisation (Raman spectroscopy, TEM and AFM), electrical properties (4‐point probe, Kelvin probe and cAFM), chemical purity and dopant quantification (XPS, FTIR and TGA). **Figure** **2** shows the different stages of graphitisation of organic, carbon‐rich materials. These changes can be monitored as a change in the Raman spectrum, in particular, the G, D and 2D bands, as can be seen further in **Figure** **3**. The G band (≈1580 cm^−1^) comes from the stretching of carbon–carbon bonds within the hexagonal lattice (the E_2*g*_ mode at the centre of the Brillouin zone). Because this vibration is always allowed, it appears in all graphitic materials. The D band (≈1350 cm^−1^) is linked to the breathing motion of six‐membered carbon rings near the K point of the Brillouin zone. It does not appear in perfect graphene, since it needs a defect or edge to conserve momentum. Its intensity therefore increases with disorder, making it a useful measure of defect density. The 2D band (≈2700 cm^−1^), also called G′, is the overtone of the D band. It arises from a double‐resonance process involving two phonons with opposite momentum near the K point. Unlike the D band, it does not need defects to appear, so it is always visible even in very high‐quality graphene. In monolayer graphene, it is a single sharp peak, while in multilayer graphene it becomes broader and splits due to interactions between layers. In amorphous carbon, the bands are very broad, the 2D peak usually does not onset, and the positions of the peaks also differ. In graphitic material, the full width at half‐maxima (FWHM) of the G and 2D bands becomes much sharper. For few‐layer graphene, interpreting the deconvolution of the G, D, and 2D bands allows for quality assessment.

### 1.2 Timeline of Graphene Fabrication Methods

Although the term “graphene” gained prominence in the early 2000s, theoretical interest in its properties dates back to the mid‐20th century. For instance, Wallace predicted in 1947 its unique band structure, and Semenoff in 1984 described the massless Dirac fermion behaviour of electrons in graphene.^[^ ^53^, ^54^ ^]^ Since its first isolation by Geim and Novoselov in 2004, graphene has driven advances in a wide range of fields, from electronics to nanotechnology.^[^ ^1^, ^3^, ^5^ ^]^

The past two decades have allowed for the development of advanced synthesis techniques. Current graphene fabrication procedures can be divided into various groups, including exfoliation (physical, chemical, or mechanical etc.), decomposition of organic materials (catalytic or non‐catalytic), chemical reduction of graphene oxide (GO) into reduced GO (rGO) or epitaxial growth (CVD).^[^ ^17^ ^]^ Graphene fabrication methods are usually segregated into bottom‐up and top‐down approaches, as was done by Yan, Nashath, Chen, Manickam, Lim, Zhao, Lester, Wu, and Pang and Wu, Wang, and Komvopoulos.^[^ ^17^, ^23^ ^]^ For the purpose of this review, the graphene fabrication techniques were segregated into bulk graphene fabrication (exfoliation), film growth (CVD, PVD) and direct graphene writing (with laser, electrons etc.) as illustrated in Figure 1.

#### 1.2.1 Graphene Fabrication from Bulk Materials

Fabrication of graphene often entails the breakdown of bulk graphite into individual graphene layers or small stacks, which distinguishes it markedly from bottom‐up approaches that synthesise graphene from molecular precursors or carbon‐rich materials.

Among bulk graphene fabrication strategies, exfoliation is predominant and encompasses mechanical, chemical, and liquid‐phase methods. **Table** **1** contains a summary of the key exfoliation techniques used to fabricate bulk graphene. The most iconic example is mechanical exfoliation via the “Scotch tape method,” first used by Novoselov et al. to isolate pristine monolayer graphene, an achievement that later earned the Nobel Prize in Physics (2010).^[^ ^1^, ^2^ ^]^ Although this method remains the benchmark for producing defect‐free monolayers, its lack of scalability limits its industrial applicability.^[^ ^55^ ^]^

**Table 1:** Summary of exfoliation techniques used to fabricate bulk graphene.

Exfoliation	Quality	Applications	Pros	Cons	Refs.
*Scotch tape method*	Pristine monolayers, large domains, minimal defects	Fundamental research, transistors, quantum devices	Highest quality graphene, ideal for research	Low yield, not scalable, manual process	^[^ ^1^, ^2^ ^]^
*LPE*	Small flakes, variable defects, few‐layer graphene	Coatings, composites, inks, energy storage	Scalable, cost‐effective, solution‐processable	High defect density, solvent contamination	^[^ ^59^, ^60^, ^61^, ^62^, ^63^ ^]^
*Chemical graphene oxide reduction*	High defect density, oxidized, reduced conductivity	Bulk graphene oxide, energy applications, membranes	Large‐scale production, inexpensive	Reduces electrical properties, requires post‐treatment	^[^ ^64^, ^65^, ^66^ ^]^
*Electrochemical*	Moderate defect density, few‐layer graphene	Flexible electronics, supercapacitors	Environmentally friendly, scalable	Difficult thickness control, lower quality than mechanical	^[^ ^67^, ^68^, ^69^ ^]^
*Intercalation‐assisted*	Layer‐by‐layer exfoliation, lower defects	Battery electrodes, printed electronics	Selective exfoliation, controlled quality	Requires specialized chemicals, limited scalability	^[^ ^70^, ^71^ ^]^

In contrast, chemical exfoliation introduces intercalating agents, such as acids or alkali metals, to expand the interlayer spacing within graphite, followed by sonication to yield graphene sheets.^[^ ^56^, ^57^, ^58^ ^]^ This approach, often used in the reduction of graphene oxide (GO), is scalable but typically introduces a high density of structural defects, necessitating further post‐treatment.

Liquid phase exfoliation (LPE), another scalable route, disperses graphite in solvents under sonication to overcome van der Waals interactions.^[^ ^59^, ^60^, ^61^ ^]^ The surface energy of the solvent must be carefully matched with that of graphene to maintain stable dispersions and prevent reaggregation.^[^ ^62^, ^63^ ^]^

While these exfoliation techniques have facilitated the integration of graphene into commercial products, they still suffer from critical limitations, including poor control over domain size, number of layers, and defect density. Moreover, patterning of graphene fabricated using these methods is nearly exclusively possible by lithography, lift‐off or precise transfer.

#### 1.2.2 Film Growth

Graphene may also be synthesised directly on a substrate, a process commonly known as bottom‐up growth. Graphene synthesis via bottom‐up methodologies primarily involves the incorporation of carbon atoms into the graphene lattice through surface‐catalysed chemical processes.

A widely adopted method is chemical vapour deposition (CVD), in which gaseous carbon sources like methane or acetylene disintegrate on catalytic metallic surfaces such as copper or nickel, facilitating the production of high‐quality monolayer graphene. This technique offers substantial control over the layer count, crystallinity, and domain size of the graphene films produced.^[^ ^17^ ^]^

Some new approaches in CVD also implement polymer‐to‐graphene conversion, in which a thin film of a polymer (most often PMMA) is deposited onto a substrate from solution.^[^ ^72^, ^73^ ^]^ The film is then converted into graphene with the help of elevated temperature (> 800 °C), application of reducing agent (H_2_ and Ar gas mix) and catalysts (Cu, Ni, Cr^[^ ^74^ ^]^).

Factors such as the temperature of the substrate, the deposition rate, and the type of metal catalyst employed play roles in determining the quality and number of graphene layers formed. For example, Wu, Wang, and Komvopoulos indicate that Cu substrates are conductive to the growth of single‐layer graphene due to their low carbon solubility, while nickel tends to produce multiple layers through carbon dissolution and precipitation upon cooling.

Epitaxial growth on SiC substrates yields high‐quality, wafer‐scale graphene with excellent crystallinity and minimal defects, but it is hindered by its high cost and limited scalability due to substrate constraints and the need for ultra‐high temperatures (>1200 °C).^[^ ^75^ ^]^ Pyrolysis of carbon‐rich materials, particularly polymer precursors, provides a facile, transfer‐free route toward direct graphene formation on various substrates, and it can be spatially confined for patterning, but it suffers from limited structural control and a higher degree of disorder, making it less suitable for electronic‐grade applications.^[^ ^76^, ^77^ ^]^ Collectively, while epitaxy ensures quality, CVD balances quality and scalability, and pyrolysis offers simplicity and integration versatility at the expense of structural precision.

### 1.3 Graphene Processing and Patterning Limitations

The commercialisation of graphene‐based electronic devices has been hindered by a series of fabrication and scalability challenges. Chief among these is the reliance on conventional lithographic processes, which often involve multiple steps, such as masking, etching, and transfer, which introduce defects, contamination, or alignment issues, particularly when aiming for nanoscale precision or heterogeneous integration.^[^ ^80^ ^]^ Moreover, high‐performance device fabrication commonly requires the synthesis of uniform large‐area monolayer graphene via CVD, a process constrained by high temperatures, specific substrate compatibility, and limited patterning capabilities.^[^ ^72^, ^81^ ^]^ These limitations collectively reduce throughput and increase production costs.

Numerous attempts have been made to improve the processability of graphene, one of which involves poly(acrylonitrile) (PAN) as a transfer medium.^[^ ^82^ ^]^ The study investigates the use of PAN as a transfer medium for wafer‐scale graphene. The authors found that PAN can effectively serve as a transfer medium, simultaneously facilitating the encapsulation and transfer of large‐area graphene films. This approach addresses common issues in graphene transfer processes, such as contamination and mechanical damage, thereby improving the quality and scalability of graphene‐based applications.^[^ ^82^ ^]^ Transferred graphene would still need to be patterned in conventional ways.

An innovative technique called Thermal, Electrical, and Water Assisted Reaction (TEAWAR) facilitates the transformation of polymethyl methacrylate (PMMA) and similar organic residues into graphene.^[^ ^83^ ^]^ The TEAWAR process leverages the synergistic effects of thermal energy, electrical stimulation, and water mediation to achieve this conversion. This approach offers a novel pathway for graphene synthesis, potentially enhancing the efficiency and sustainability of graphene production from polymeric materials.

Attempts to create graphene‐rich inks for 3D printing were reviewed by Jiang, Guo, Liu, Xu, and Gao. The review outlines recent developments in 3D printing of graphene‐based materials and their applications in energy storage and conversion devices and discusses the extrusion‐based direct ink writing technique, emphasising the rheological behaviour of graphene oxide (GO) dispersions and strategies for preparing printable GO inks. The review highlights how 3D printing enables the design of advanced electrode architectures, potentially improving the performance of energy storage devices.^[^ ^8^ ^]^ This methodology, however, does not align with the scope of this review and graphene‐ink‐based additive manufacturing methods will not be considered further.

This raises the question of how the concept of “direct writing” of graphene should be understood. Within the scope of this review, the term direct designates processes that circumvent any post‐deposition treatments, such as annealing, whereas writing denotes an additive manufacturing–like approach in which a precursor undergoes a chemical transformation during pattern formation. Furthermore, such a direct‐write methodology inherently demands high spatial resolution. Accordingly, a genuine graphene direct‐write technique would necessitate the use of a sufficiently small probe capable of enabling additive manufacturing with precise spatial control.

**Figure 4:**
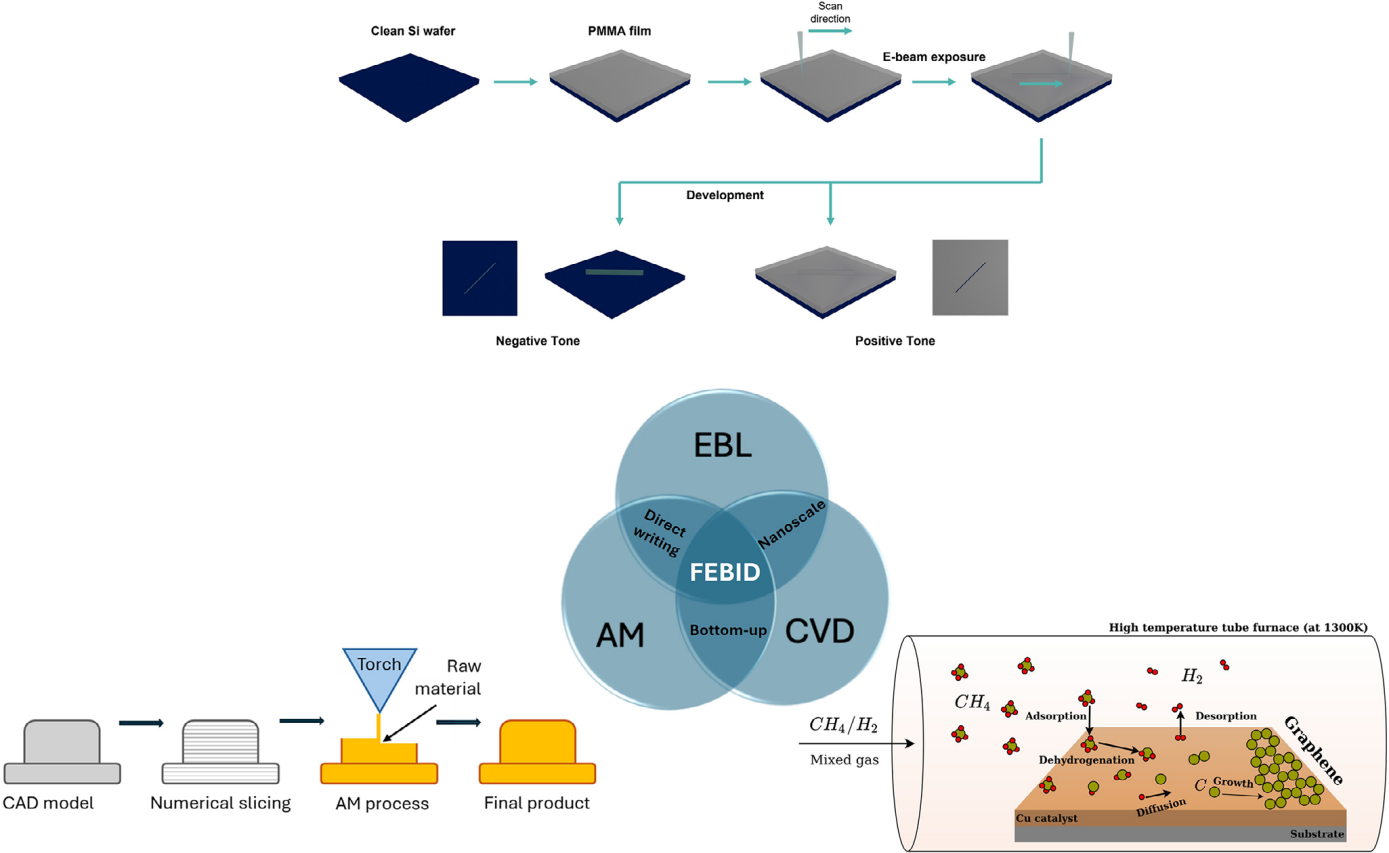
Venn diagram showing various advantages shred between EBL, CVD, Additive manufacturing (AM) with electron beam induced deposition sharing all these. The processes are also summarised as diagrams. The CVD diagram was adapted under the terms of the CC BY 4.0 license.^[^ ^84^ ^]^

## 2 Focused Electron Beam Induced Deposition (FEBID)

### 2.1 Fundamentals of Electron‐Material Interactions

Focused electron beam‐induced deposition (FEBID) relies on intricate interactions between the electron beam and, for example, reactive gas, thin films, and even substrates, to transform materials and deposit it directly and in controlled mannner on a nanoscale. These processes resemble the ones occurring during electron beam lithography (EBL) (see **Figure** **4**) which has similar modus operandi, thus both of these research areas use similar approaches and terminology. as. Both primary electrons (PE) and secondary electrons (SE), as well as substrate‐mediated effects, play critical roles in these material transformations. PEs, typically generated at voltages between 0.1 and 30 kV, penetrate the substrate and lose energy via inelastic collisions, generating cascades of secondary electrons (SEs) with energies usually below 50 eV. These SEs are largely responsible for the surface‐localised dissociation of precursor molecules in FEBID due to their highly localised energy deposition. Higher energy PEs result in broader interaction volumes, whereas lower acceleration voltages confine the effect near the surface. Backscattered electrons (BSEs), more prominent on high‐atomic‐number substrates such as Cu or Ni, contribute to a lateral broadening of the deposition area.^[^ ^85^ ^]^ In parallel, electron‐induced ionisation leads to radiolysis, where precursor bonds can be cleaved to form radicals and volatile species such as CO, CO_2_, and H_2_O. The amount of electron radiation required to achieve the material transformation, called dose (D) is defined as:

(1) $$D=\frac{1}{A}\int_{0}^{t}i\left(t\right)\;dt$$

where A is area, t is time, and i is current. Typically in EBL, this dose is a representation of the minimal value at which the resist (usually polymer) transforms by bond cleavage, or cross‐linking. At high doses, this residual carbon can transition into amorphous or sp^2^‐rich carbon domains, setting the stage for graphene‐like structures. For conversion of polymer to graphene (P2G) these values are expected to be much higher than for EBL as the reaction end is expected not as initial transformation but rather as complete graphitisation. In FEBID, this value indicates the amount of radiation needed to convert the gaseous precursor and deposit the solid onto a substrate e.g. by the removal of ligands from the precursor which makes it volatile or bond cleavage and formation of radicals that reassemble and deposit as solid.^[^ ^86^, ^87^ ^]^ Although knock‐on displacement typically requires electron energies above 80–100 keV—beyond the range of standard SEM‐based EBID—lighter atoms like hydrogen and oxygen may still be displaced, contributing to the purification of the deposit. Substrate properties also significantly influence the process: conductive substrates (e.g., Cu, Ni, doped Si) dissipate charge and often catalyse graphitisation, whereas insulating substrates (e.g., SiO_2_, glass) can accumulate charge, leading to beam distortion. Variable pressure conditions with inert gases may help mitigate charging without interfering chemically. Substrate atomic number affects BSE yield and, consequently, the spatial extent of deposition. Importantly, catalytic substrates such as Cu can aid carbon atom diffusion and reorganisation, facilitating higher‐quality graphene growth. Collectively, the combination of SE‐driven dissociation, radiolytic decomposition, selective knock‐on displacement, and catalytic substrate interaction would drive the success of EBID in forming graphene nanostructures.

### 2.2 Separation into Graphitizable and Non‐Graphitizable Materials

The separation of graphitizable and non‐graphitizable materials is a fundamental concept in carbon material science, particularly relevant to the fabrication of graphene and other graphitic structures.^[^ ^88^, ^89^, ^90^ ^]^ Upon thermal treatment, carbon‐rich precursors exhibit divergent structural transformations depending on their molecular structure and bonding characteristics. Graphitizable materials usually have high H:C ratios and low O(+N+S)/C ratios and produce local molecular orientations during the graphitisation process.^[^ ^89^, ^90^ ^]^ Non‐graphitizable materials, on the other hand, are rich in N, O and S, and even at graphitisation temperatures do not form LMOs and remain turbostatic.^[^ ^89^, ^90^ ^]^

Graphitizable materials, such as certain pitches, polycyclic aromatic hydrocarbons, and certain polymers, undergo structural reordering upon heating, eventually forming crystalline graphene layers with high stacking order and extended lateral dimensions. This transition is facilitated by the absence of cross‐linking side groups and a high degree of aromaticity, allowing carbon atoms to rearrange into the thermodynamically favourable graphitic lattice. In contrast, non‐graphitizable materials, PMMA, possess a cross‐linked, amorphous structure due to the presence of heteroatoms and functional groups such as hydroxyls. These groups hinder the alignment and growth of ordered graphene layers, resulting in materials that remain largely disordered even at elevated temperatures. The distinction between these two classes of materials could be particularly significant in the context of electron beam processing, where the structural predisposition of the precursor determines the feasibility and quality of graphitisation, with direct implications for the electronic, optical and mechanical properties of the resulting carbon films.^[^ ^88^, ^91^ ^]^

A molecular dynamics study by Francas, Martin, Suarez‐Martinez, and Marks also provides valuable insight into the precursor dependence of the graphitisation process.^[^ ^92^ ^]^ The authors demonstrated that screw dislocations represent the dominant annealable defects in graphitizable carbons, where their removal facilitates progressive structural ordering. In contrast, persistent saddle‐shaped defects and associated negative curvatures hinder the development of long‐range order. Screw dislocations were also observed in the graphitisation process of poly(vinyl chloride) (PVC) using TEM, which further confirmed that these features play a key role in the structural reorganisation pathway during graphitisation.^[^ ^93^ ^]^

Although these studies do not directly address e‐beam induced graphitisation, they underline that the precursor material and its defect landscape critically determine the achievable crystallinity.^[^ ^92^, ^93^ ^]^ For electron‐beam induced direct writing approaches, this implies that the beam dose and interaction volume must be carefully tuned not only to break bonds but also to facilitate defect annihilation. Moreover, while bulk graphitisation proceeds rapidly at extreme temperatures (> 2500 °C), electron‐beam processing typically operates at lower effective thermal budgets, which can limit the extent of defect removal and long‐range ordering. Hence, understanding the balance between defect creation and annihilation is essential for scalable and controllable nanopatterning of high‐quality graphene.

It seems that the vast majority of research on polymer‐to‐graphene conversion is influenced by the research on electron beam lithography on PMMA as a resist material.^[^ ^94^ ^]^

### 2.3 Polymer‐To‐Graphene (P2G) Conversion Using Electron Beam

**Figure 5:**
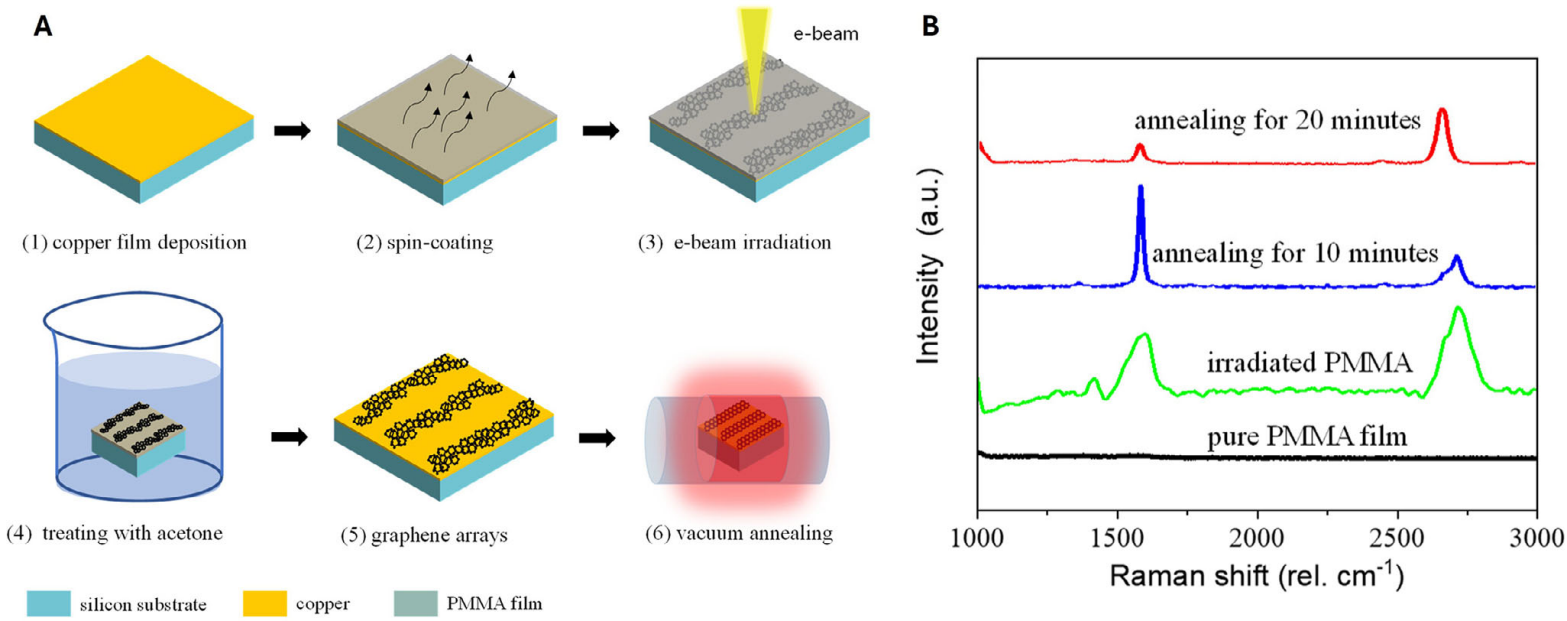
A) The process flow diagram of the e‐beam lithographic process for the fabrication of graphene nanostructures on copper substrate. Low‐quality graphene was formed at step 3, and high quality graphene at step 6. B) Raman spectra of the sample at the different preparation stages. Reproduced under the terms of the CC BY 4.0 license.^[^ ^94^ ^]^ Copyright 2021, The Authors.

EBID enables the direct transformation of polymeric precursors into graphenic materials through a combination of radiolytic and thermal effects.^[^ ^95^ ^]^ Upon e‐beam irradiation, the polymer matrix undergoes ionization and bond scission, producing free radicals that recombine to form a cross‐linked, carbon‐rich network as per **Figure** 5A. Simultaneously, dehydrogenation and deoxygenation expel heteroatoms such as hydrogen, oxygen, and nitrogen, enriching the sp^2^‐carbon content. Polymers like PMMA, polystyrene, PAN, polyimides, and phenolic resists (e.g., SU‐8 or AZ5214) display distinct responses under EBID, influenced by their molecular structure. For instance, PMMA carbonises into amorphous features, while PAN undergoes electron‐induced cyclization, mimicking thermal stabilisation and yielding nitrogen‐doped graphenic carbon.^[^ ^94^, ^96^, ^97^ ^]^ Polyimides, rich in heteroatoms, can form porous, conductive graphite foams under high‐dose irradiation.^[^ ^98^ ^]^ Beyond chemical changes, the e‐beam also induces localised heating via inelastic scattering, facilitating pyrolysis and bond reconfiguration. Substrate effects further influence this process; conductive metals like Cu or Ni not only dissipate heat but also catalyse dehydrogenation and induce planarisation. Post‐irradiation annealing, typically at 300 to 800 °C in inert or reducing atmospheres, significantly enhances structural ordering and conductivity, driving the reorganisation of disordered carbon into graphitic domains. While EBID alone often yields amorphous or turbostratic carbon, sustained irradiation or thermal treatment promotes graphitisation, especially in the presence of catalytic substrates. The final material typically comprises nanocrystalline graphite or a mosaic of small graphene domains, with resistivities ranging from 10^−4^ to 10^−3^ Ω cm, which are sufficient for use in microelectrodes, sensors, and other functional carbon nanostructures. Thus, EBID offers a controllable, lithography‐compatible method for bottom‐up fabrication of graphenic nanomaterials, with tunable properties governed by precursor chemistry, electron dose, and thermal conditions.

### 2.4 Electron Beam‐Induced Modifications in SAMs

**Figure 6:**
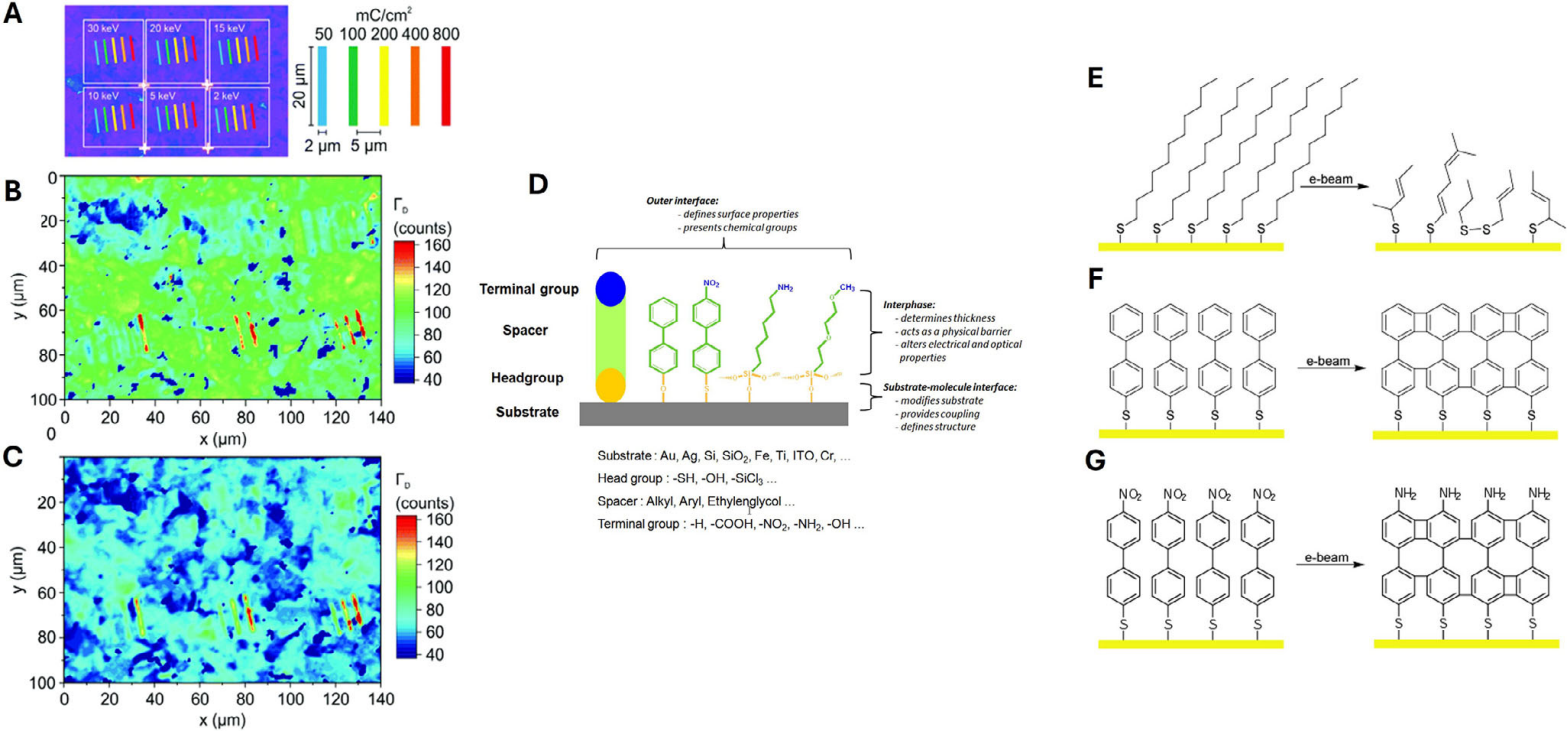
A) Scheme of the electron irradiation of oxo‐G flakes on a SiO_2_ (300 nm)|Si wafer. The colour code indicates the dose ranging from 50 to 800 m C cm^−2^ for the beam energies between 2 and 30 keV. B) Raman map of a film of oxo‐G flakes mapping the full‐width at half‐maximum (Γ_*D*_) of the Raman D peak after e‐beam irradiation of different energy and doses. Red: broad D peaks (low quality, amorphous), blue narrow D peaks (better quality). C) Raman map of the same area as in (B) of reduced oxo‐G after FEBIP. Introduced areas of increased defect densities (red area) are permanent. Figures A– C reproduced under the terms of the CC BY‐NC 3.0 license.^[^ ^99^ ^]^ Copyright 2017, The Authors. D) Schematic representation of the constituents and their functions in a self‐assembled monolayer (SAM) 2. Electron beam induced modification of SAMs: E) aliphatic SAMs; F)aromatic SAMs; G)aromatic SAMs with nitro termination. Figures D– G are reproduced with permission. Turchanin and Gölzhäuser Copyright 2012, Elsevier.

Electron beam‐induced modifications in self‐assembled monolayers (SAMs) offer a powerful and versatile approach for tailoring surface chemistry and nanoscale patterning with high spatial resolution. Upon exposure to low‐energy electron irradiation, typically with an acceleration voltage 0.1 to 5 keV, SAMs undergo a variety of physico‐chemical transformations, including bond cleavage, desorption, cross‐linking, and reorientation of molecular backbones (see **Figure** **6**D–G).^[^ ^101^ ^]^ In particular, SAMs featuring carboxylic acid (CA) anchoring groups on coinage metal substrates such as silver have demonstrated pronounced susceptibility to electron‐induced reactions.^[^ ^101^ ^]^ These modifications are driven by electron‐stimulated cleavage of the carboxylate–metal bond, leading to partial or complete desorption of molecules, followed by the formation of a carbonaceous residue via cross‐linking of the remaining organic fragments.^[^ ^101^ ^]^ The presence of aromatic backbones in CA‐based SAMs enhances the propensity for cross‐linking due to delocalised π‐electrons, enabling the conversion of the monolayer into a robust, insoluble carbon nanomembrane.^[^ ^101^ ^]^ Furthermore, electron irradiation can induce significant conformational and orientational changes in the SAMs, such as reorientation or disordering of the molecular packing.^[^ ^101^ ^]^ The graphitisation at relatively low temperatures (≈730 K) of electron beam irradiated SAMs was also observed with Raman and TEM.^[^ ^100^ ^]^ These effects are highly dependent on the molecular structure, packing density, and energy and dose of the electron beam. Such controlled modifications are increasingly exploited in applications ranging from lithography and surface patterning to the fabrication of chemically and mechanically stable nanostructures.^[^ ^100^, ^101^, ^102^ ^]^

### 2.5 Gaseous Precursors

**Figure 7:**
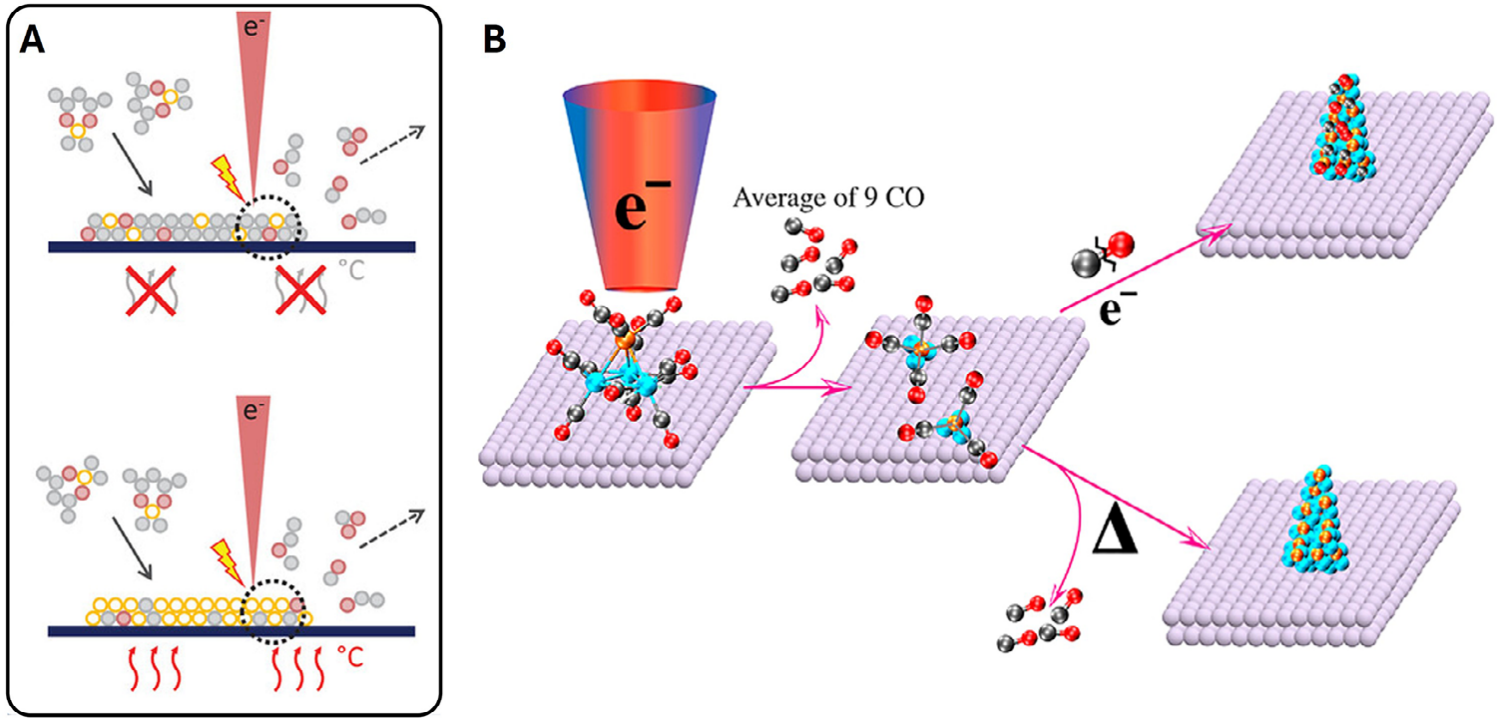
A) Schematic representation of the area‐selective autocatalytic film growth. A seed layer is deposited using a focused electron beam to locally decompose the precursor on a substrate (top) at room temperature (30 °C) or (bottom) at elevated substrate temperatures. Figure reproduced with permission.^[^ ^103^ ^]^ Copyright 2024, Wiley‐VCH GmbH. B) Schematic illustrating different stages of ligand cleavage in the fragmentation of HFeCo_3_(CO)_12_ and two subsequent effects leading to i) a composite formation by further electron bombardment or ii) complete ligand stripping by a thermally activated process step resulting in a pure FeCo_3_ alloy. Reproduced with permission.^[^ ^104^ ^]^ Copyright 2018 American Chemical Society.

Electron beam‐induced graphene growth can also be performed using carbonaceous gases such as ethylene, acetylene, or phenanthrene being introduced into the electron microscope chamber. The focused electron beam dissociates these molecules, depositing carbon‐rich material, eventually graphene. A similar process of electron beam induced dissociation of gaseous material and subsequent deposition of carbon‐rich material was represented in **Figure** **7**. Graphene growth using TEM from VOC contamination has also been previously reported.^[^ ^105^ ^]^ To our knowledge, this has not yet been achieved in a SEM setting.

In 2002, Kiyohara, Takamatsu, and Mori used a mix gas of H_2_ and CH_4_ to directly write diamond onto a Si(100) wafer.^[^ ^106^ ^]^ They used a in‐house modified TOPCON DS‐130S equipped with a sub‐chamber where all gases were injected as shown in Figure 10. They found that the optimal concentration of CH_4_ in the chamber was ≈1 % for formation of sp^3^ carbon structures. Above that regime, the formation of more sp^2^ carbon structures took place. This knowledge suggests a potential direction for direct write graphene from the H_2_ and CH_4_ mix gas. The paper also highlights the use of Tempilstick as a method to determine local temperature changes.^[^ ^106^ ^]^

**Figure 8:**
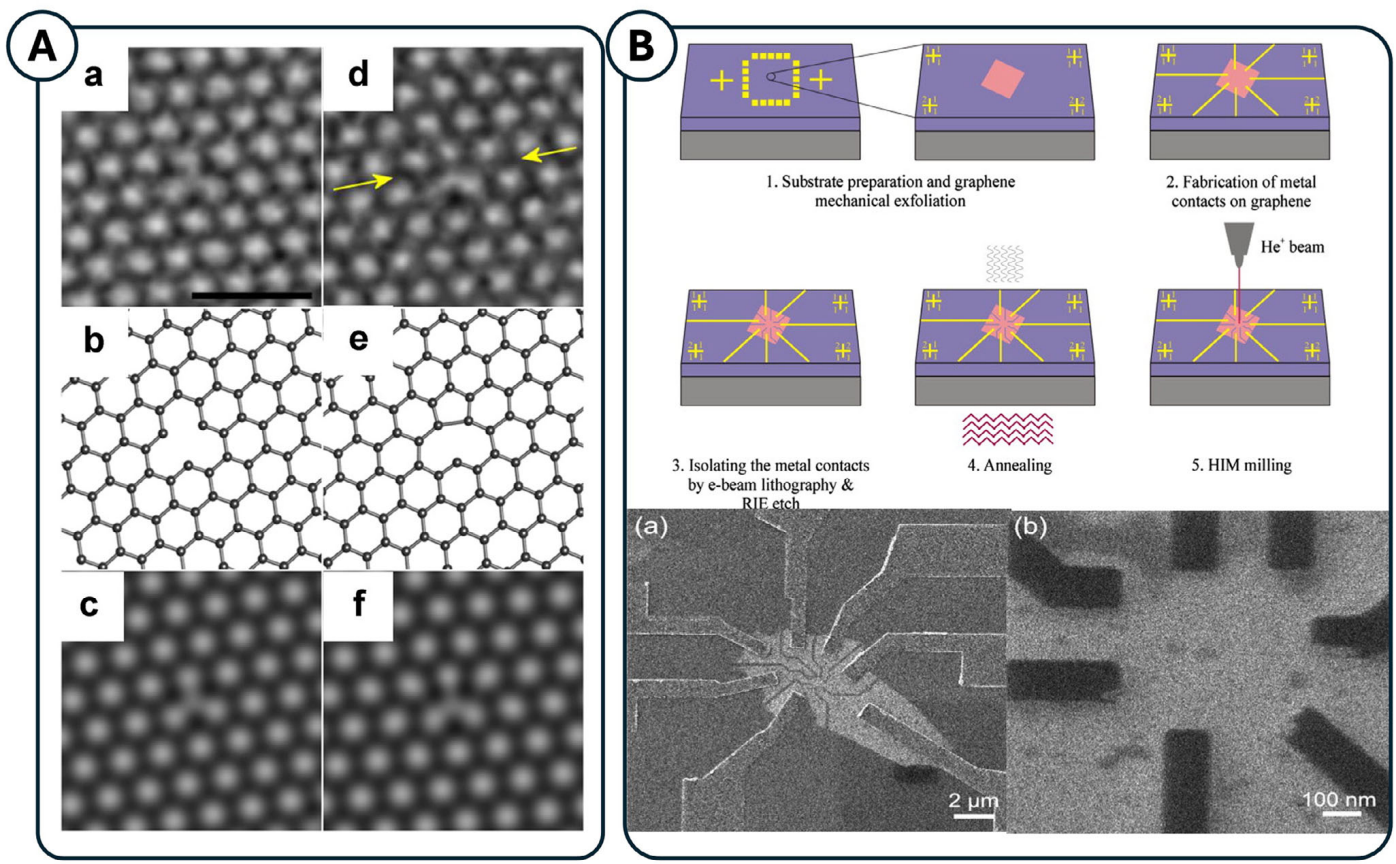
A) (a) Smoothed AC‐TEM image of the symmetric monovacancy, s‐MV, with three edge C atoms. (b) DFT‐calculated atomistic model and (c) multislice TEM image simulation corresponding to the structure in (a). (d) Smoothed AC‐TEM image of the reconstructed monovacancy, r‐MV, with one edge C atom. Yellow arrows indicate the zigzag axis containing the reconstruction. (e) DFT‐calculated atomistic model and (f) multislice TEM image simulation corresponding to the structure in (e). Scale bar corresponds to 1 nm in all panels. Figure is reproduced with permission.^[^ ^21^ ^]^ Copyright 2013 American Chemical Society. B) Schematic illustration of the steps involved in the fabrication of GDQD by HIM milling.(a) HIM SE image of a monolayer graphene flake with metal contacts and RIE‐formed isolation lines. (b) HIM SE image of the etched isolation lines (dark lines in the figure) which are forming an area of ≈500 nm × 420 nm on the flake that was left intact for the final HIM patterning step. Figure adapted with permissions.^[^ ^121^ ^]^ Copyright 2014 Elsevier.

**Figure 9:**
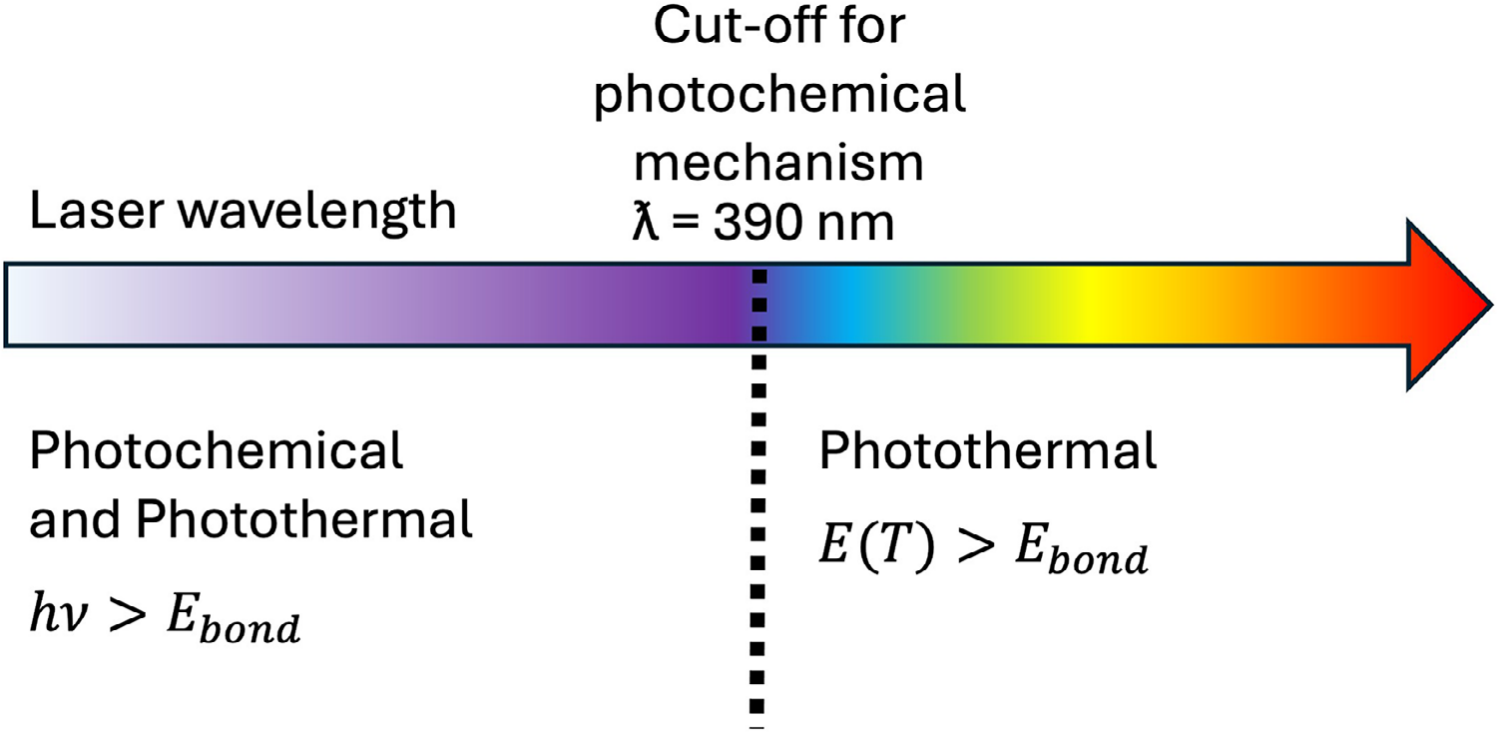
Mechanism of laser‐induced deposition. A schematic diagram showing that photochemical processes occur only at higher energy radiation (>390 nm) while photothermal processes happen in the regimes from IR to UV.^[^ ^126^, ^127^ ^]^

**Figure 10:**
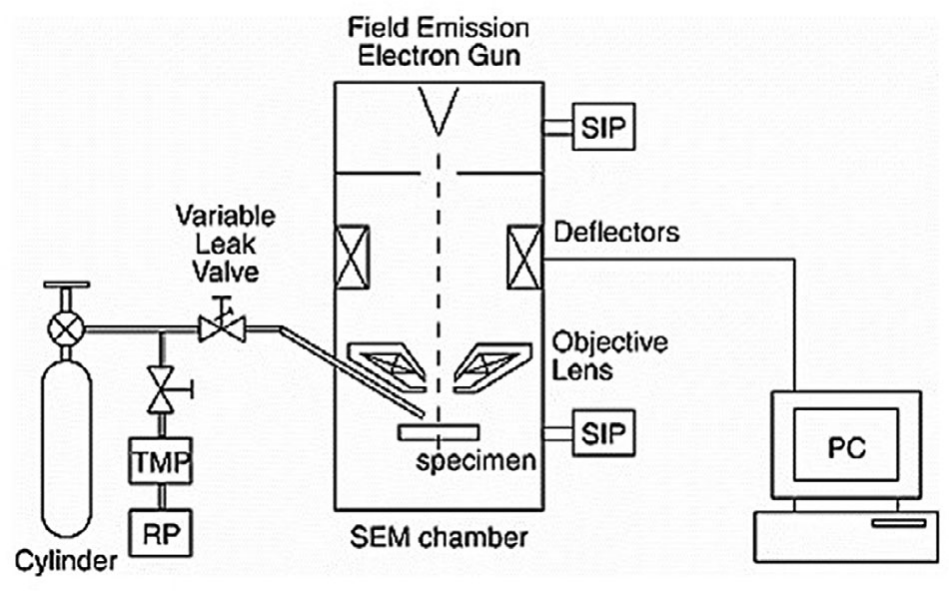
Schematic drawing of a 30 kV FEG‐SEM equipped with dual‐gas introduction system for fabrication of the nanorods by EBID with a mixture gas of iron carbonyl and ferrocene. Figure reproduced with permission.^[^ ^150^ ^]^ Copyright 2004, Elsevier.

Other gaseous precursors can also be used to nucleate^[^ ^103^ ^]^ or directly write^[^ ^107^, ^108^ ^]^ carbon‐rich metal‐based nanostructures such as Pt nanoparticles,^[^ ^108^, ^109^ ^]^ Nb and CoFe nanostructures.^[^ ^107^ ^]^ The carbon content depends on multiple variables including the precursor used (e.g. incomplete ligand stripping, CO cleavage), residual hydrocarbons in the chamber atmosphere, sample preparation methods, beam settings etc., and most of them are in a porous state.^[^ ^104^, ^107^, ^110^ ^]^ Although most of the carbon content in these structures is amorphous there is a potential for further conversion to graphene and creation of attractive composite materials, for example, for sensing applications.^[^ ^108^, ^110^, ^111^, ^112^, ^113^ ^]^

### 2.6 Limitations and Challenges in FEBID

Deposits obtained by FEBID often suffer from poor material quality due to incomplete precursor decomposition, leading to carbon‐ and oxygen‐rich nanogranular structures that require extensive post‐processing to achieve functional purity. Despite its maskless versatility and 3D writing capability, the method is constrained by the limited range of volatile precursors and their complex fragmentation pathways. There is still insufficient research regarding in situ deposition of graphene using reactive gases, or direct P2G conversion under a focused electron beam. Moreover, the outcome strongly depends on substrate properties and residual chamber gases, which introduce variability and compromise reproducibility, thereby partially limiting compatibility with large‐area and semiconductor manufacturing workflows.

## 3 Focused Ion Beam (FIB)

### 3.1 Fundamentals

Irradiation with focused ion beam (FIB) induces a range of modifications in graphene, depending on the ion species, energy, dose, and ambient environment.^[^ ^114^ ^]^ At low ion doses, collisions between ions and carbon atoms primarily generate isolated point defects, such as vacancies or bond distortions, without significantly altering the overall morphology.^[^ ^115^ ^]^ As the dose increases, these defects agglomerate into nanopores, and at sufficiently high doses, heavy‐ion bombardment amorphises the carbon lattice and can eventually perforate or fully sputter the graphene sheet.^[^ ^116^ ^]^ In addition to structural damage, certain ion species, such as gallium (Ga^+^) commonly used in FIB, can be implanted into graphene, causing strain, increasing defect density, and altering electronic properties such as the work function.^[^ ^114^ ^]^ Lighter ions such as helium (He^+^) or neon (Ne^+^), while less likely to dope the lattice due to their chemical inertness, create finely controlled physical defects and allow for high‐resolution patterning.^[^ ^117^ ^]^ Reactive ions such as nitrogen (N^+^), boron (B^+^), or fluorine (F^+^) can covalently dope the lattice; for instance, low‐energy N^+^ irradiation introduces pyridinic and graphitic nitrogen sites before vacancy formation dominates at higher fluences.^[^ ^118^ ^]^ Ion irradiation can also induce localised stress and lattice distortions, with heavier ions causing bond rehybridization (from sp^2^ to sp^3^) and occasionally forming adatoms or clusters (e.g., Ga or Cu) that further modify electrical and chemical behaviour.^[^ ^119^ ^]^ Comparatively, Ga^+^ ions are highly efficient at sputtering (50 % sputtering yield) but simultaneously implants the Ga into graphene lattice doping the material, while He^+^ and Ne^+^ offer superior resolution and minimal chemical alteration (97 % of He^+^ ions are transmitted at high energies with no destructive effects), making them more suitable for precision nanopatterning.^[^ ^120^ ^]^ Overall, the interplay between physical displacement and chemical interaction defines the structural and electronic transformations of graphene under FIB.^[^ ^118^, ^119^, ^120^ ^]^

### 3.2 Graphene Milling

Numerous studies have been conducted on the application of FIB for the milling of graphene and localised doping. Kalhor, Boden, and Mizuta used a helium ion microscope (HIM) to fabricate double quantum dots of graphene from mechanically exfoliated graphene supported on Si wafer patterned with a combined study of EBL and reactive ion etching (RIE) for CMOS applications. HIM images of this example can be found in **Figure** **8**.^[^ ^121^ ^]^ HIBM not only allowed for superior imaging resolution, but also allowed for graphene milling at the nanometre scale in scanning mode, which is often called helium ion beam milling (HIBM).^[^ ^122^ ^]^ Schmidt, Muruganathan, Kanzaki, Iwasaki, Hammam, Suzuki, Ogawa, and Mizuta used a similar approach of HIBM to fabricate graphene nanoribbons with the first report of a functional device with characterization of electrical properties.^[^ ^123^ ^]^ The research group was also able to achieve a graphene nanomesh with tunable conductance using HIBM.^[^ ^124^ ^]^ HIBM was also previously reported as fabrication method for nanoporous graphene for biosensing, in this case DNA.^[^ ^125^ ^]^

### 3.3 Limitations and Challenges in FIB

FIB and HIBM provide unmatched spatial control for graphene nanopatterning, yet both techniques face fundamental limitations. Conventional FIB with Ga^+^ ions induces significant structural damage and chemical contamination through ion implantation, leading to defects, strain, and degraded electronic performance. Lighter ion sources such as He^+^ or Ne^+^ (in HIBM) reduce implantation but still generate vacancies, nanopores, and edge disorder at high doses. Both approaches remain intrinsically serial and non‐scalable, restricting their use to prototyping rather than wafer‐scale fabrication. Additionally, the high cost and limited availability of helium ion systems further hinder their practical adoption. Consequently, while FIB and HIBM are powerful research tools for high‐resolution graphene structuring, their damage profiles and throughput constraints limit their suitability for large‐scale, device‐grade applications.

## 4 Laser‐Assisted Patterning

### 4.1 Fundamentals

Laser‐assisted graphene deposition fundamentally relies on two distinct physical mechanisms: photothermal and photochemical effects; whose dominance is determined by the photon energy of the laser source (See **Figure** 9). These two regimes play a crucial role in determining the quality, morphology, and degree of graphitisation of the resulting material.^[^ ^17^, ^126^, ^127^ ^]^

In the photothermal regime, where the photon energy is below ≈3.2 eV (wavelengths longer than 390 nm, i.e. visible and infrared lasers), the formation of graphene is driven primarily by localised heating. The carbon‐rich precursor (often polymers such as polyimide) absorbs the laser energy, which is then converted to heat. This thermal energy initiates pyrolysis, fragmenting the polymer and leading to carbonisation once the temperature exceeds a critical threshold (≈2700 K). At this point, molecular fragments reorganise into sp^2^‐bonded carbon networks characteristic of graphene. This regime follows Arrhenius‐type behaviour: Higher laser power or slower scanning speeds enhance local temperature and graphitisation.^[^ ^72^, ^73^ ^]^

In contrast, the photochemical regime becomes relevant with ultraviolet (UV) lasers possessing photon energies above ≈3.2 eV (wavelengths < 390 nm). In this regime, bond dissociation (e.g. of C–O, C = O, or C–N) can occur via direct photoexcitation, independent of thermal effects. UV photons can break chemical bonds directly at lower fluences, thereby reducing the temperature required for carbonisation. However, as laser fluence increases, photothermal effects again become significant, resulting in a mixed photochemical–photothermal mechanism. This hybrid mechanism enables more efficient and precise graphene formation, as observed in excimer laser ablation studies.

At sufficient laser fluence and energy density, the irradiated precursor not only carbonises but evolves into a porous graphene foam due to rapid gas evolution (CO, CO_2_, H_2_O, etc.). This outgassing results in microporosity and a foam‐like structure with vertically orientated, few‐layer graphene sheets, known as laser‐induced graphene (LIG). Although this structure is typically not atomically flat, its large surface area and exposed edges offer distinct advantages for sensing and energy applications.

The ultrafast, non‐equilibrium nature of laser‐induced graphene formation can trap structural defects. These are commonly observed as strong D‐bands in Raman spectra. Nonetheless, post‐treatment methods, such as rapid Joule flash heating, have demonstrated effectiveness in healing these defects, improving crystalline order, and enhancing electrical conductivity.

The surrounding atmosphere and pressure play a decisive role in the quality and composition of LIG. Early demonstrations established that polyimide could be directly converted to graphene in ambient air at atmospheric pressure using a CO_2_ laser, yielding a scalable and maskless route to porous conductive films.^[^ ^128^ ^]^ However, irradiation in air also leads to the incorporation of oxygen and nitrogen functional groups, as reactive species from the atmosphere participate in bond scission and subsequent doping processes.^[^ ^129^ ^]^ Inert or reducing atmospheres improve the outcome: when CO_2_ laser scribing was performed under argon or Ar/H_2_ flows at room pressure, higher quality graphene with fewer defects was obtained, as hydrogen assisted in removing oxygen functionalities and promoted sp^2^ carbon reconstruction.^[^ ^130^ ^]^ Comparative studies with UV and CO_2_ lasers further confirmed that ambient conditions favoured nitrogen‐doped LIG, whereas argon atmospheres yielded more pristine graphene‐like domains.^[^ ^129^ ^]^ Atmosphere not only governs defect incorporation but also electrical performance. LIG produced in ambient air typically shows sheet resistances under 40 Ω *sq* ^−1^, sufficient for microsupercapacitors and sensors, but with higher defect densities from oxygen and nitrogen incorporation.^[^ ^128^ ^]^ By contrast, inert or reducing atmospheres yield graphene with improved crystallinity, lower sheet resistance (≈ 10 Ω *sq* ^−1^), and enhanced conductivity.^[^ ^130^ ^]^ Although low‐pressure or vacuum environments are not required for the scribing itself, mild vacuum treatment post‐fabrication can be applied to remove residual volatiles and stabilise the graphene surface. Thus, while LIG is intrinsically compatible with atmospheric‐pressure processing, the choice of gaseous environment enables tailoring of defect density and functional group incorporation.

### 4.2 Laser Type Influence

Laser‐assisted graphene synthesis can be achieved using various types of lasers, offering distinct processing advantages based on their wavelength, pulse duration, and interaction with carbon‐rich precursors. Infrared CO_2_ lasers (10.6 µm), with photon energies (≈0.117 eV) far below typical bond dissociation energies, operate via a strongly photothermal mechanism.^[^ ^128^, ^130^ ^]^ These deliver deep substrate heating, generating thick, porous few‐layer graphene with a relatively low defect density, especially on polymers such as polyimide. Due to their long wavelength and thermal diffusion, CO_2_ lasers are limited in spatial resolution ≈100–200 µm), but are ideal for rapid, large‐area graphene writing in ambient conditions, as demonstrated by Lin, Peng, Liu, Ruiz‐Zepeda, Ye, Samuel, Yacaman, Yakobson, and Tour and Ye, Chyan, Zhang, Li, Han, Kittrell and Tour.^[^ ^128^, ^129^, ^130^ ^]^ Visible lasers like 532 nm (green) are also predominantly photothermal, with moderate polymer absorption and intermediate feature resolution (≈50 µm).^[^ ^131^, ^132^ ^]^ These have been employed in laser chemical vapour deposition (LCVD) using metal substrates, such as Ni, to catalyse graphene growth, as shown by Chen, Ren, Gao, Liu, Pei, and Cheng, and in direct laser writing on PI films by Huang, Feng, Yin, Zhou, Shen, Wang, and Luo.^[^ ^131^, ^132^ ^]^

The near‐UV regime (e.g. 355 nm), laser energy (≈3.5 eV) begins to approach carbon bond dissociation energies, enabling partial photochemical interactions. This results in enhanced absorption in polymers and finer spatial resolution (≈30–50 µm), although care must be taken to avoid substrate ablation. These lasers can produce more defective graphene, often with weaker 2D Raman bands, as reported by Wang, Wang, Bakhtiyari, and Zheng and Liu and Chen.^[^ ^129^, ^133^ ^]^ Deep UV excimer lasers (e.g. 248 nm) extend this trend, offering strong photochemical activation (≈5 eV per photon) and allowing bond scission without substantial heating. Used in inert atmospheres to prevent oxidation, deep UV lasers can write graphene with high spatial precision (<20 µm), especially useful in microelectronic applications, as demonstrated by Singleton, Paraskevopoulos, and Irwin, Yu, Gai, Liu, Chen, Bian, and Huang. (2020).^[^ ^80^, ^126^, ^134^ ^]^

Femtosecond (fs) lasers, operating in the visible or near‐IR/UV range, exploit ultrashort pulses (on the order of 10^−15^ s) to induce non‐linear effects like multiphoton ionisation and microplasma formation. This confines energy deposition spatially and temporally, minimising thermal diffusion and allowing extremely fine feature writing (≈10 µm or less). Femtosecond lasers have been used to produce hierarchically porous graphene with high surface area with degree of doping, as reported by Liu and Chen, although the resulting material often contains more edge defects (high D‐band intensity) unless it is post‐annealed.^[^ ^133^ ^]^ Lastly, blue diode lasers (405–450 nm), commonly found in low‐cost engravers, provide an accessible photothermal tool for LIG on absorptive substrates, although their lower power and weaker absorption often yield lower graphene quality. These are more prevalent in hobbyist or educational settings, with few high‐quality scientific reports to date.^[^ ^129^, ^130^, ^132^ ^]^

### 4.3 Limitations and Challenges in LIG

Despite its appeal as a scalable and maskless direct‐write approach, LIG suffers from a number of intrinsic limitations that hinder its integration into high‐performance nanoelectronics. The process is highly substrate dependent, as it typically requires polyimide or other specific polymers that undergo local carbonisation, restricting material versatility and compatibility with standard semiconductor platforms.^[^ ^128^, ^135^ ^]^ The resulting graphene is often porous and foam‐like in morphology, which benefits certain applications such as supercapacitors or sensors but poses challenges for achieving continuous, high‐mobility conductive films. Furthermore, control over the number of layers and crystallinity remains limited, with partial graphitisation and the persistence of structural disorder reducing the electronic quality compared to CVD‐grown films.^[^ ^128^ ^]^ Another critical drawback lies in the spatial resolution; even when using tightly focused beams, the thermal diffusion associated with laser processing limits the achievable feature size to the micrometre scale, well above the nanoscale precision required for advanced integrated devices.^[^ ^135^ ^]^ Finally, the process is energetically intensive and sensitive to laser parameters such as wavelength, pulse duration, and power density, making reproducibility across different platforms non‐trivial. Collectively, these issues restrict LIG's role to niche applications where porosity and scalability are advantageous, while its utility in logic electronics or beyond‐silicon computing remains limited.

## 5 Other Techniques

Several additional techniques enable the nanopatterning and doping of graphene, among which scanning probe microscopy (SPM) plays a particularly prominent role. Graphene nanopatterning, as revealed through STM studies, demonstrates remarkable versatility, with features engineered from the atomic scale up to hundreds of nanometres^[^ ^136^, ^137^ ^]^ Defect engineering affords direct‐write precision, wherein individual vacancies or hydrogen chemisorption sites can be introduced with atomic accuracy, enabling highly controlled functionalisation.^[^ ^136^, ^138^, ^139^ ^]^ Strain engineering, by contrast, induces periodic ripples, nanobubbles, and moiré patterns, offering both top‐down lithographic and bottom‐up self‐assembly routes to structured domains.^[^ ^136^, ^140^, ^141^, ^142^, ^143^ ^]^ Moreover, quantum confinement within engineered quantum dots and resonators allows graphene to emulate artificial atoms and molecules, thereby opening routes toward quantum devices.^[^ ^136^, ^144^, ^145^, ^146^, ^147^ ^]^ Taken together, these approaches establish nanopatterning as a powerful means of tailoring graphene's electronic and topological properties.^[^ ^136^, ^143^, ^148^ ^]^

AFM‐related techniques have likewise been applied for the nanopatterning of graphene. Dynamic Plowing Lithography (DPL) has proven particularly effective, enabling direct mechanical modification of graphene through cutting, displacement, and even rolling of layers under applied forces up to ≈68 µN.^[^ ^149^ ^]^ Grooves with depths of 1–4 nm and widths of 40–50 nm have been reproducibly achieved, demonstrating controlled nanoscale modification.^[^ ^149^ ^]^ Beyond simple trenches, more intricate features such as graphene nanoislands, concentric rings, and even patterned words (e.g., “NANO”) have been fabricated, underscoring the versatility of the method. Crucially, DPL circumvents the need for pre‐patterned metallic contacts or resist layers, offering a maskless and resist‐free route to graphene nanostructuring.^[^ ^149^ ^]^ Overall, AFM‐based DPL highlights the potential of mechanical scanning probe lithography as a flexible, direct‐write nanofabrication platform for graphene electronics.^[^ ^149^ ^]^

## 6 Conclusion

FIBM, inparticular HIBM, while demonstrating considerable potential for the direct writing of various materials, is better suited for milling and doping applications in the context of graphene, particularly through the use of reactive ions such as Ga^+^. However, its utility in direct graphene writing remains limited due to structural damage and ion contamination, which compromise the integrity of the sp^2^ network. In contrast, laser‐assisted patterning emerges as a powerful tool for microscale structuring, offering the advantages of high throughput and the possibility of in situ Raman spectroscopy, a capability particularly beneficial for real‐time monitoring of structural and electronic changes in graphene during processing. Among these techniques, FEBID appears to hold the greatest promise in terms of direct‐write capability, material selectivity, and threshold‐based control over deposition processes. However, concerns persist about the quality of the graphene‐like material deposited, particularly due to the incorporation of organic residues and low sp^2^ content in the deposited films. Despite these limitations, FEBID remains a highly tunable and versatile approach, especially when coupled with post‐deposition purification or graphitisation strategies (See **Table** 2).

**Table 2:** Comparison of direct‐write graphene techniques with conventional CVD and bulk exfoliation methods in terms of process conditions, directness, fabrication paradigm, limitations, and scalability.

Method	Process conditions	Directness	Fabrication Paradigm	Limitations	Scalability
FEBID	SEM environment, carbonaceous gas or polymer precursor, vacuum, controlled electron dose	Quasi‐direct (requires precursor, sometimes annealing)	Bottom‐up, additive nanofabrication	Low sp^2^ content, contamination, precursor dependence	High resolution (nm), but poor throughput and wafer‐scale scalability
FIB & HIBM	High‐energy ion irradiation (Ga^+^, He^+^, Ne^+^), vacuum chamber	Indirect (mainly subtractive, defect engineering)	Subtractive, doping possible	Damage, sputtering, ion implantation	Excellent spatial control, but serial and non‐scalable
LIG	Ambient or controlled gas, IR–UV lasers, photothermal or photochemical conversion of polymers	Semi‐direct (requires precursor film)	Additive (polymer carbonisation)	Low resolution (10 ‐ 100 µm), defect‐rich graphene	Highly scalable, rapid, low‐cost
STM & AFM	UHV or controlled atmosphere; nanometre‐scale tip; local bias or force‐induced bond breaking/formation	Direct (tip induces bond rearrangements or local oxidation/reduction)	Bottom‐up or subtractive (nanoscale writing/modification)	Extremely slow, limited area, tip wear, demanding conditions	Ultimate resolution (atomic scale), but not scalable
CVD	High T (≈1000 °C), CH_4_, H_2_, or Ar gases, catalytic substrates (Cu, Ni)	Indirect (requires transfer or patterning)	Bottom‐up film growth	Transfer defects, substrate limitations	Wafer‐scale and industrially scalable
Bulk	Sonication or chemical exfoliation in solvents, mild heating	Non‐direct (graphene flakes, patterned later)	Top‐down disintegration of bulk	Defects, flake size variability, solvent contamination	Highly scalable (inks, composites), poor device‐grade quality

### 6.1 Challenges to be Addressed

One of the principal challenges in the domain of direct graphene writing lies in the ambiguity of what constitutes true “directness.” Current approaches such as P2G conversion via electron or laser irradiation are only semidirect in nature, owing to their reliance on a prior deposition of a carbon‐rich polymer precursor. Furthermore, these methodologies frequently require a post‐irradiation thermal annealing step, often in inert or reducing environments, which further undermines the claim of directness. For a process to be truly direct, it would require in situ graphitisation of ambient or gaseous carbonaceous species, such as volatile organic compounds in the atmosphere or by forming a gas, without any precursor layer. Compounding this are issues of resolution: due to the optical transparency of monolayer and few‐layer graphene, visualisation and pattern fidelity assessment remain nontrivial, demanding sophisticated techniques such as SEM, Raman mapping, or conductive AFM. Moreover, precise alignment of the written features, particularly in multilayer or device‐integrated architectures, necessitates advanced stage control and fiducial referencing systems.

Emerging techniques such as FEBID, FIBM (in particular HIBM), and laser‐assisted direct writing present promising pathways, yet each carries limitations in terms of throughput, contamination, or control over crystallinity, thus warranting further investigation and refinement. Laser‐induced graphene (LIG), for instance, offers scalability, ambient operation, and precursor versatility, but suffers from intrinsic constraints. Its resolution is limited to the micrometre scale due to thermal diffusion, and the resulting graphene is typically porous and foam‐like, unsuitable for high‐mobility channels. The process is also strongly substrate‐dependent, with polyimide being the dominant precursor, limiting applicability across semiconductor platforms. Furthermore, incomplete graphitisation and high sensitivity to laser wavelength, power density, and pulse duration impede reproducibility and integration into standard microelectronic workflows.

FIB based nanopatterning, including HIBM, provides unrivalled spatial precision for defect engineering, nanopore sculpting, and nanoribbon fabrication. Nonetheless, these methods inevitably introduce structural damage. Ga^+^ FIB implantation leads to doping, vacancy generation, and strain, while lighter ions such as He^+^ and Ne^+^ mitigate implantation but still degrade lattice order at higher doses. The technique is also inherently serial and non‐scalable, rendering it unsuitable for wafer‐level device fabrication despite its value for prototyping and fundamental studies. In addition, the high capital and operational costs of He ion systems restrict broader accessibility.

STM and AFM methods, offer atomic‐scale resolution and the ability to engineer localised defects, quantum dots, and functional group modifications. These approaches provide unique opportunities to study quantum and topological effects in graphene. However, they remain fundamentally constrained by the low throughput and serial nature of scanning probe techniques. Their reliance on highly specialised instrumentation means that they are best positioned as tools for exploratory device concepts and fundamental studies, rather than practical large‐area integration.

For FEBID, from a hardware perspective, the implementation of direct graphene writing within SEM equipped with a thermionic electron gun, environmental chamber, gas injection system (GIS), a heating stage, and a picoamperometer introduces a complex interplay of operational challenges and instrumental constraints (see **Figure** **10**). The thermionic gun, while robust and widely accessible, offers relatively lower brightness and spatial coherence compared to field emission sources, potentially limiting the achievable resolution in patterning and increasing interaction volumes, complicating control over lateral feature sizes and edge sharpness. The environmental chamber, though indispensable for in situ gas‐phase reactions or pressure‐regulated processes, introduces complications in electron scattering and beam stability, thereby requiring precise regulation of chamber pressure and gas composition to mitigate image degradation and ensure beam fidelity.

The GIS, a critical enabler for introducing carbonaceous precursors or forming gas, can pose contamination risks if not finely calibrated, potentially leading to undesirable carbon deposition in non‐targeted areas or even optical components such as electron lenses etc. Additionally, the incorporation of a heating stage, essential for post‐irradiation conversion or catalytic enhancement, demands thermal isolation and robust material compatibility to prevent off‐gassing or structural warping under prolonged heating cycles.

Importantly, the BSD detector, often located close to the sample chamber, is vulnerable to contamination from volatilised precursors or beam‐induced sputtering in gas‐rich environments. Prolonged exposure to reactive gases, especially under elevated temperatures and electron bombardment, can degrade detector sensitivity or induce fouling on sensitive components, including scintillators or photomultiplier tubes. Proper shielding, controlled gas flow paths, and regular maintenance schedules become imperative to protect detector integrity and overall instrument longevity. These multifaceted constraints underscore the necessity of carefully orchestrated hardware configurations and operational protocols to enable reliable, reproducible, and truly “direct” graphene writing in SEM platforms.

### 6.2 Future Work

Emerging directions in FEBID and LIG for graphene patterning reflect a confluence of technological innovation and sustainability considerations. Low‐dose precursor‐to‐graphene strategies are being explored for patterning on flexible substrates, such as those used in paper‐based electronics, enabling lightweight and disposable graphene‐based devices. Hybrid workflows that integrate electron beam lithography with chemical vapour deposition (CVD) offer promising avenues for spatially selective doping, enhancing functionality at the nanoscale. The implementation of machine‐learning algorithms to optimise beam patterning parameters is poised to significantly improve precision, throughput, and reproducibility simultaneously being able to avoid proximity effects, drifts etc. Concurrently, the development and deployment of environmentally benign precursors are gaining traction, driven by the imperative for sustainable and less hazardous e‐beam processing protocols. Furthermore, the field is witnessing a growing interest in the identification and classification of graphitizable materials suitable for electron beam‐induced deposition (EBID) of graphene. This necessitates a revised framework to guide the rational selection of precursors based on their structural evolution under electron irradiation.

## Conflict of Interest

The authors declare no conflict of interest.
